# Determination of *F_v_*/*F_m_* from Chlorophyll *a* Fluorescence without Dark Adaptation by an LSSVM Model

**DOI:** 10.34133/plantphenomics.0034

**Published:** 2023-03-30

**Authors:** Qian Xia, Hao Tang, Lijiang Fu, Jinglu Tan, Govindjee Govindjee, Ya Guo

**Affiliations:** ^1^ Key Laboratory of Advanced Process Control for Light Industry, Ministry of Education, Jiangnan University, Wuxi 214122, China.; ^2^ Department of Biomedical, Biological and Chemical Engineering, University of Missouri, Columbia, MO 65211, USA.; ^3^ Center of Biophysics and Quantitative Biology, Department of Biochemistry and Department of Plant Biology, University of Illinois at Urbana-Champaign, Urbana, IL 61801, USA.

## Abstract

Evaluation of photosynthetic quantum yield is important for analyzing the phenotype of plants. Chlorophyll *a* fluorescence (ChlF) has been widely used to estimate plant photosynthesis and its regulatory mechanisms. The ratio of variable to maximum fluorescence, *F_v_*/*F_m_*, obtained from a ChlF induction curve, is commonly used to reflect the maximum photochemical quantum yield of photosystem II (PSII), but it is measured after a sample is dark-adapted for a long time, which limits its practical use. In this research, a least-squares support vector machine (LSSVM) model was developed to explore whether *F_v_*/*F_m_* can be determined from ChlF induction curves measured without dark adaptation. A total of 7,231 samples of 8 different experiments, under diverse conditions, were used to train the LSSVM model. Model evaluation with different samples showed excellent performance in determining *F_v_*/*F_m_* from ChlF signals without dark adaptation. Computation time for each test sample was less than 4 ms. Further, the prediction performance of test dataset was found to be very desirable: a high correlation coefficient (0.762 to 0.974); a low root mean squared error (0.005 to 0.021); and a residual prediction deviation of 1.254 to 4.933. These results clearly demonstrate that *F_v_*/*F_m_*, the widely used ChlF induction feature, can be determined from measurements without dark adaptation of samples. This will not only save experiment time but also make *F_v_*/*F_m_* useful in real-time and field applications. This work provides a high-throughput method to determine the important photosynthetic feature through ChlF for phenotyping plants.

## Introduction

Photosynthesis is the source of food, energy, fiber, and oxygen for all living organisms including humans. Evaluation of photosynthetic quantum yield is important for analyzing plant phenotypes; however, the research of current plant phenomics is often limited to external geometry features. When the chloroplasts in plants and algae absorb sunlight, pigments, mainly chlorophyll molecules, in the light-harvesting pigment protein (antenna) complexes are excited and the absorbed energy is transferred to photosystem II (PSII) and photosystem I (PSI) reaction centers [1]. The absorbed light energy is used mostly for photosynthesis but is partly dissipated in the form of chlorophyll *a* fluorescence (ChlF) or heat [2]. Background on the various steps of photosynthesis is available in several publications [3,4].

Environmental or plant physiological changes that affect PSII lead to changes in ChlF, which can be used as a fast, sensitive, and a nondestructive indicator of the status of PSII [5,6]. Analysis of ChlF changes is one of the most powerful and widely used techniques to study the effects of various types of stress on the photosynthetic process [7–9]. At present, ChlF is widely used as a probe for not only PSII but overall photosynthesis [10], photosynthetic systems [11], photochemistry and heat dissipation [12], several photosynthetic reactions [13], and photoinhibition [14]. Furthermore, it is used to monitor different types of abiotic stress [15], including drought [16], heat [17,18], environmental pollution [19], nutrient status [20], and plant phenotyping [21]. ChlF measurement can serve as a plant physiological variable related to photosynthesis in phenotypic analysis. Advances in optical phenotyping (including that by ChlF) of cereal crops have been summarized by Sun et al. [22].

Although ChlF has been used for many purposes, as mentioned above, the interpretation of ChlF measurement is quite complex. A very important feature derived from the ChlF induction curve is *F_v_*/*F_m_* [23], which allows us to provide information on effects of carbon metabolism and has been successfully used as a sensitive indicator of the photosynthetic performance of plants [24]. To determine the *F_v_*/*F_m_* ratio, dark adaptation is needed to open all the PSII reaction centers, and only then can the minimal fluorescence (*F_o_*) be measured. (For a discussion on the timing for measuring *F_o_*, see the study by Padhi et al. [25].) After excitation with strong continuous light, most, if not all, the reaction centers are closed, and thus, ChlF reaches a maximum value (*F_m_*). The difference, *F_v_* = *F_m_* − *F_o_*, is referred to as the variable fluorescence. The ratio, *F_v_*/*F_m_* = (*F_m_* − *F_o_*)/*F_m_*, reflects an intrinsic PSII efficiency and measures the quantum yield of the primary PSII photochemistry in dark-adapted photosynthetic samples [26,27]. *F_v_*/*F_m_* has been successfully used as an indicator of plant photosynthetic performance [28]. It has also been used to obtain information on photoinhibition induced by abiotic stress [29]. The *F_v_*/*F_m_* can also reflect the severity of plant phenotypic diseases, and it is an important indicator of plant stress. Rousseau et al. [21] focused on phenotyping by analyzing *F_v_*/*F_m_* images, and their results showed that there was a clear strong difference between the infected tissues and the healthy tissues. Zhou et al. [30] used ChlF in the phenotypic analysis of faba beans (*Vica faba* L.) under both cold and heat stress and found that *F_v_*/*F_m_* is a very effective parameter in detecting the damage by low and high temperatures to PSII; further, they identified high-temperature-tolerant broad bean genotypes. Therefore, *F_v_*/*F_m_* can be used as a physiological marker for phenotyping.

Before measuring *F_o_*, it is necessary to dark-adapt a plant sample for 15 to 30 min [31] or even longer [32]. This dark-adaptation process is time-consuming. Far-red light, absorbed mainly by PSI, might be used to speed up the oxidation of the reduced plastoquinone (PQ) pool and thus suppress the measured *F_o_*, i.e., *F_o_*′ (minimum ChlF intensity in the light-adapted state) increase, and this method is often applied following dark adaptation. It is thus desirable to find a method to determine *F_v_*/*F_m_* from ChlF measurement without dark adaptation. The exact relationship between ChlF with dark adaptation and that without dark adaptation is complex and has not yet been established. By using contemporary computational methods, this hidden relationship can be explored to determine accurate *F_v_*/*F_m_* from ChlF measurement without dark adaptation, but this has not yet been done by any research group.

Artificial intelligence methods have been widely used to identify hidden relationships in many fields. Using these methods to analyze ChlF data can identify complex relationships in plant responses to stresses [33]. Tyystjärvi et al. [34] have identified species of crops and weeds by analyzing ChlF induction curve with an artificial neural network method. This method has been used to identify plant species by analyzing ChlF induced by different types of illumination [35]. Furthermore, Goltsev et al. [36] have constructed and trained an artificial neural network by using photoinduced prompt ChlF, delayed ChlF, and 820-nm modulated reflection signal (measuring PSI) to identify changes in the photosynthetic activity in bean leaves during drying. Yao et al. [37] have applied kinetic ChlF and multi-color fluorescence imaging technology for phenotypic analysis of *Arabidopsis* drought stress response, and, from it, they have successfully classified *Arabidopsis* under different drought stress levels by a support vector machine (SVM). Artificial intelligence methods may be potentially used to find the hidden relationship between the *F_v_*/*F_m_* and ChlF measurement, without any dark adaptation of plants, but by using a general learning strategy (i.e., a mathematical method), so that *F_v_*/*F_m_* under dark adaptation can be predicted from ChlF measurement without dark adaptation.

In our present study, a least-squares SVM (LSSVM), an artificial intelligence method, was used to determine *F_v_*/*F_m_* from ChlF measurement without dark adaption for multiple plant species and conditions, which allows one to save tremendous amount of experimental time and provides an important feature for plant phenomics.

## Materials and Methods

### Plant samples

Eight sets of experiments with a total of 7,231 samples were performed on 6 plant species (*Oryza sativa* L. [rice], *Camellia japonica*, *Euonymus japonicus Thunb*, *Osmanthus* sp, *Cerasus lannesiana* var. *speciosa*, and *Capsicum annuum*). These plant species are under different drought stress, ambient growth temperature, growing seasons, and measured environments. Details are described below in the order they were done from the summer of 2019 until the winter of 2021 for different plant species, described below.

#### *Rice* (*Oryza sativa* L.)

The first set of experiments was conducted on rice plants (*Oryza sativa* L.) under 4 different drought stress conditions. Rice plants were taken with roots from a production field in Jiangsu, China, in the early mornings, during the growing season in the summer of 2019, when the ambient temperature was ~28 °C. To reduce the effects of variations in moisture in different samples, during ChlF measurements, the roots of the plants were completely immersed in water for at least 2 h. Then, the roots were placed in 20% polyethylene glycol for different durations (0, 1, 2, and 4 h) of treatment to achieve different levels of drought stress or physiological state [38]. The number of samples of rice plants without drought or with drought treatment for 1, 2, and 4 h was 1,335, 1,093, 1,322, and 1,146, respectively. The temperature during ChlF measurement was between 30 and 36 °C, and the ambient photosynthetic photon flux density (PPFD) was between 3 and 7 μmol photons m^−2^ s^−1^.

#### Camellia japonica and Euonymus japonicus Thunb

The second set of experiments was carried out on Japanese Camellia (*Camellia japonica*) leaves, using 314 samples. The third set of experiments was done on leaves of *Euonymus japonicus Thunb*, also using 314 samples. Both *Camellia japonica* and *Euonymus japonicus Thunb* were grown on the campus of Jiangnan University (Wuxi, China). Leaves from these 2 plants were picked in the mornings in April 2021 and were transferred immediately to the laboratory for measurements. To reduce the effect of variations in the water condition, the sampled leaves of the second and the third sets of experiments were floated on water for at least an hour. The temperature during ChlF measurement was ~23 °C and the ambient PPFD was ~5 μmol photons m^−2^ s^−1^.

#### *Osmanthus sp. and Cerasus lannesiana* var*. speciosa*

The fourth and the fifth sets of experiments were carried out on intact plants in the wild field, using leaves of *Osmanthus* sp. with 237 samples and those of *Cerasus lannesiana* var*. speciosa* with 335 samples. These plants in the fourth and the fifth experiments were grown naturally on the campus of Jiangnan University (Wuxi, China). The ChlF data of the fourth and the fifth experiments were collected at the end of July 2021, the ambient temperature was ~33 °C, and the ambient PPFD was between 58 and 1,960 μmol photons m^−2^ s^−1^.

#### Capsicum annuum

The sixth set of experiments was performed on attached leaves of *Capsicum annuum.* Here, 356 samples were tested in the field, which were grown in a greenhouse in Wuxi, China. The ChlF data were collected at the beginning of August 2021. The temperature was between 36 and 40 °C, and the ambient PPFD was between 58 and 1,770 μmol photons m^−2^ s^−1^ during measurements in the greenhouse.

#### Camellia japonica and Osmanthus sp.

The seventh and eighth experiments were carried out on intact plants on the campus of Jiangnan University (Wuxi, China), which included leaves of *Osmanthus* sp. with 379 samples and of *Camellia japonica* with 400 samples. These experiments were done in December 2021; the ambient temperature was between 8 and 15 °C, and the ambient PPFD was between 78 and 1,380 μmol photons m^−2^ s^−1^. Table 1 shows all plant samples and experiment specifics.

### Instrumentation and measurements

The ChlF parameter *F_v_*/*F_m_* (ratio of variable to maximum fluorescence) was measured under 2 conditions: with and without dark adaptation of the leaves. The illumination condition without dark adaptation means that the plant leaves are not dark-adapted before the ChlF measurement. The leaves were measured without dark adaptation, and then they were measured in dark-adapted state after dark adaptation. Twenty-minute dark adaptation was applied through dark-adaptation clips [39]. A FluorPen ChlF measurement device (Photon Systems Instruments, Drásov, Czech Republic) was used to measure ChlF transient, ChlF induction of the leaves, where O is the minimum fluorescence, J and I are inflection steps, and P is for the peak (the maximum). The illumination light intensity to excite the ChlF of leaves was set as 2,400 μmol photons m^−2^ s^−1^ for all samples.

The ambient light intensities for all our experiments were measured by a light intensity meter (VC1010A, Victor, Shenzhen, China). The light intensity read in Lux, from the measured light intensity meter, was converted to PPFD. The conversion relationships are 1 Klux = 19.5 μmol photons m^−2^ s^−1^ for daylight PPFD [40] and 1 Klux = 12 μmol photons m^−2^ s^−1^ for white fluorescent light [41]. The values of ambient light intensities in this work are only used to show that measurements were made on samples illuminated with a wide range of initial lighting conditions. Estimation errors of PPFD from Lux have no effect on the conclusion of this work.

### Development of an LSSVM model

An SVM maps high-dimensional data from an input space to a feature space through a nonlinear mapping process. LSSVM is an extension of SVM; it uses inequality constraints instead of equality constraints and the sum of squared-error loss function as the “experience loss” to transform a problem into a linear one. In this work, an LSSVM model was employed to map the relationship between the ChlF induction feature *F_v_*/*F_m_* with and without dark adaptation of the photosynthetic samples. The LSSVM regression equation is:

$$f\left(x\right)=w^{T}\varphi \left(x\right)+b$$ (1)

where *x* is the ChlF response without dark adaptation, *f*(*x*) is the corresponding output, *φ*(*x*) is a nonlinear mapping function that maps *x* to a high-dimensional feature space, *w* is a weighting vector, and *b* is a bias variable. Based on the principle of structural risk minimization, the function becomes:

$$f\left(x\right)=\sum_{i=1}^{m}a_{i}K\left(x,x_{i}\right)+b$$ (2)

where *K* is a kernel function, *a_i_* is the Lagrangian multiplier, *i* is an integer, and *m* is the number of samples in a training dataset. According to the Mercer condition, the kernel function can be written as:

$$K\left(x_{i},x_{j}\right)=\varphi {\left(x_{i}\right)}^{T}\varphi \left(x_{j}\right)\;i,j=1,2,\ldots ,m$$ (3)

The following radial basis function was used as the kernel function in our research:

$$K\left(x,x_{i}\right)=\exp\left\{-\frac{{\left\|x-x_{i}\right\|}^{2}}{2\tau^{2}}\right\}$$ (4)

where τ represents the parameter of the Gaussian radial basis kernel function.

For the training dataset {(*x_i_*, *y_i_*), *i* = 1, 2, …, *m*}, *x_i_*∈R^m^ represents the input of the *i*-th training sample (ChlF measured without dark adaptation), *y_i_*∈R is the target value of the *i*-th training dataset (*F_v_*/*F_m_* measured with dark adaptation), and *m* is the number of samples in the training dataset.

For the testing dataset {(*X_i_*, *Y_i_*), (*i* = 1, 2, …, *n*)}, *X_i_* is the input of the *i*-th test sample (ChlF measured without dark adaptation), *Y_i_* is the real target value of the *i*-th test data sample (*F_v_*/*F_m_* measured with dark adaptation), and *n* is the number of samples in the test dataset. *X_i_* is fed to the trained LSSVM model (Eq. 2) to calculate the corresponding predicted *F_v_*/*F_m_* value, and the *i*-th predicted *F_v_*/*F_m_* value is expressed as *YY_i_* (*i* = 1, 2, …, *n*).

### Data normalization

To reduce the influence of differences in data magnitudes, the following zero-mean normalization method (Z-score normalization) was used to normalize both the ChlF signal data without dark adaptation and the *F_v_*/*F_m_* target values with dark adaptation so that both were in the same order of magnitude:

$$Z=\frac{x-\mu }{\sigma }$$ (5)

where μ denotes the mean and σ is the SD of the original data *x*, and *Z* represents standard normal distribution.

The predicted *F_v_*/*F_m_* values from the model were denormalized to their original scale for testing and evaluation.

### Model testing and evaluation

To evaluate the performance and generalization ability of the model, the following metrics computed from the test samples were used to assess the predicted *F_v_*/*F_m_*: (a) root mean square error (*RMSE*); (b) correlation coefficient (*CC*); and (c) residual predictive deviation (*RPD*), as shown below in Eqs. 6 to 8.

$$\text{RMSE}=\sqrt{\frac{\sum_{i=1}^{n}{\left({\mathrm{YY}}_{i}-Y_{i}\right)}^{2}}{n}}$$ (6)

$$\mathrm{CC}=\frac{\sum_{i=1}^{n}\left(\left({\mathrm{YY}}_{i}-\bar{\mathrm{YY}}\right)\left(Y_{i}-\bar{Y}\right)\right)}{\sqrt{\left[\sum_{i=1}^{n}{\left({\mathrm{YY}}_{i}-\bar{\mathrm{YY}}\right)}^{2}\right]\left[\sum_{i=1}^{n}{\left(Y_{i}-\bar{Y}\right)}^{2}\right]}}$$ (7)

$$\mathrm{RPD}=\sqrt{\frac{\sum_{i=1}^{n}{\left(Y_{i}-\bar{Y}\right)}^{2}}{\sum_{i=1}^{n}{\left({\mathrm{YY}}_{i}-Y_{i}\right)}^{2}}}$$ (8)

where *YY_i_* is the predicted *F_v_*/*F_m_* value of the *i*-th test sample, *Y_i_* is the true *F_v_*/*F_m_* value of the *i*-th test sample, $\bar{Y}$ is the true *F_v_*/*F_m_* mean value of the test samples, and *n* is the number of samples in the test dataset. All these metrics measure the deviation of the predicted *F_v_*/*F_m_* values from the true values. As is commonly known, the smaller *RMSE* or the closer to unity *CC* is, the higher the prediction performance. For most applications, models with *RPD* values lower than 1.5 are considered insufficient, while models with values greater than 2.0 have good robustness [42].

In the training of the LSSVM model, a 10-fold cross-validation, and a grid optimization, was used to optimize the 2 parameters (regularization coefficient and parameter of the Gaussian radial basis kernel function) that affect the accuracy and the complexity of the model. In each of the 10 runs, 10%, 20%, …, and 90% of each sample type was randomly selected as the training dataset, and the remaining was used as the testing dataset. The average values of *RMSE*, *CC*, and *RPD* obtained in the 10 runs ($\bar{\text{RMSE}}$, $\bar{\mathrm{CC}}$, and $\bar{\mathrm{RPD}}$) were used to evaluate model performance. The LSSVM model was implemented in MATLAB 2019b (Mathworks, Inc., Natwick, MA, USA).

## Results

### Variations in *F_v_*/*F_m_* with dark adaptation and without dark adaptation

To explore the difference between different sample types of the *F_v_*/*F_m_* measured with and without dark adaptation, statistical comparisons on the *F_v_*/*F_m_* from different sample types are presented in Table 2. Values indicated with different letters in a column are significantly (*P* < 0.05) different from one another by the LSD (least signification difference) test. The *F_v_*/*F_m_* measured with and without dark adaptation show statistical differences between most different sample types and treatments, as shown below in Table 2.

### Training performance of the model for prediction of *F_v_*/*F_m_* using ChlF without dark adaptation

We note that 10%, 20%, …, and 90% of A0, A1, A2, A3, B1, B2, C, D, E, F, and G were randomly selected as the training dataset to establish the initial LSSVM model, and the remaining samples were used as the verification dataset to test the prediction performance of the established LSSVM model for *F_v_*/*F_m_* under dark adaptation. The all-rice test datasets were composed of rice samples with 4 different drought levels. All *Osmanthus* sp. test datasets were composed of *Osmanthus* sp. samples in summer and winter. The $\bar{\mathrm{CC}}$, $\bar{\text{RMSE}}$, and $\bar{\mathrm{RPD}}$ represent the average values of the *CC*, *RMSE*, and *RPD*, respectively.

LSSVM model performance evaluation indices ($\bar{\mathrm{CC}}$, $\bar{\text{RMSE}}$, and $\bar{\mathrm{RPD}}$) for determining *F_v_*/*F_m_* values of training dataset from ChlF without dark adaptation under different training dataset sample numbers are shown in Tables 3 to 5. When the training dataset sample exceeds 70%, the $\bar{\mathrm{CC}}$ of most sample types for the training dataset is greater than 0.80 in Table 3, and the $\bar{\mathrm{RPD}}$ of most sample types for the training dataset is greater than 1.5 for the training dataset in Table 5. The $\bar{\text{RMSE}}$ of different sample types for the training dataset is less than 0.016 in Table 4.

### Prediction of *F_v_*/*F_m_* using ChlF without dark adaptation on the test dataset

The test dataset results of using the LSSVM model to determine *F_v_*/*F_m_* from ChlF measured without dark adaptation under different training dataset sample numbers are presented in Tables 7 and 8. By all the measures (see Tables 7 and 8), the model, used in our research, showed strong prediction performance when the training dataset sample is more than 80% of all sample size. Under this condition, the $\bar{\mathrm{CC}}$ values for the test dataset show (Table 6) that the predicted *F_v_*/*F_m_* by the LSSVM model are nearly perfectly in most cases, correlated with the true *F_v_*/*F_m_* values, the most $\bar{\mathrm{CC}}$ values being more than 0.80. The $\bar{\text{RMSE}}\;$ values for the test dataset in Table 8 show nearly negligible differences between the predicted and the real *F_v_*/*F_m_*, the $\bar{\mathrm{RPD}}$ values of the most sample types are much greater than 2, and all $\bar{\mathrm{RPD}}$ values are greater than 1.5, which shows that the model has good robustness for the test dataset.

**Fig. 1:**
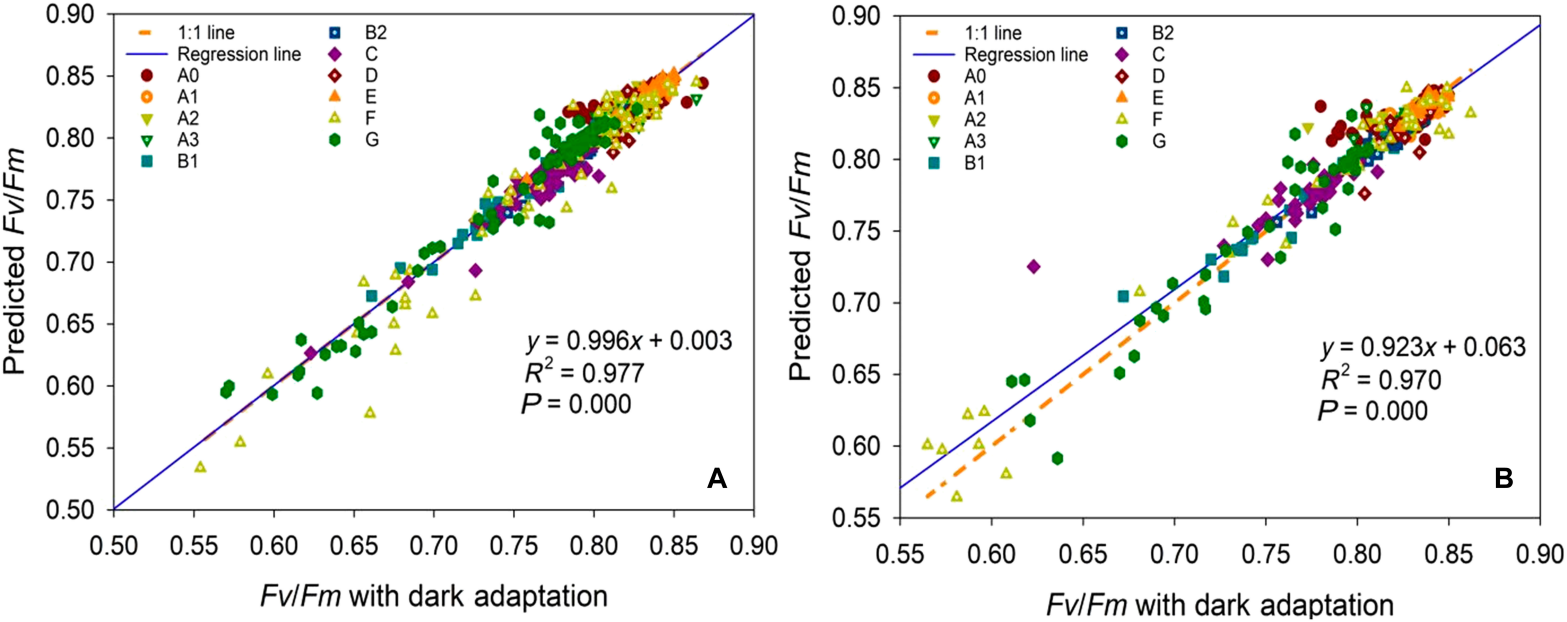
The *F_v_*/*F_m_* predictions for test dataset using the LSSVM model obtained by different training dataset sample numbers. (A) The number of training dataset is 80% of the total sample. (B) The number of training dataset is 90% of the total sample. (A0: rice without drought treatment; A1: rice with 1 h of drought treatment; A2: rice with 2 h of drought treatment; A3: rice with 4 h of drought treatment; B1: *Osmanthus* sp. in summer; B2: *Osmanthus* sp. in winter; C: *Euonymus japonicus Thunb* in the laboratory; D: *Camellia japonica* in the laboratory; E: *Capsicum annuum*; F: *Cerasus lannesiana* var*. speciosa*; G: *Camellia japonica* in wild field*.*)

Figure 1 shows a comparison of the *F_v_*/*F_m_* values predicted by the LSSVM model obtained from different training dataset sample numbers with the experimental values measured after dark adaptation for all the tested samples. It is obvious from the plots that the predicted *F_v_*/*F_m_* values by the LSSVM model match the real values of *F_v_*/*F_m_* well. To further evaluate model prediction performance, we computed a regression line to verify whether it is close to the 1:1 line. As shown in Figure 1, the fitted regression lines have small slopes and intercept errors; further, the predicted values for *F_v_*/*F_m_* almost coincide with the perfect 1:1 line for the sample types used. The data points are tightly distributed around the ideal straight line, which means that the predicted values are linearly related to the real values. The coefficient of determination (*R^2^*) values between the predicted *F_v_*/*F_m_* and the measured *F_v_*/*F_m_* values with dark adaptation is 0.970 for all plant samples, which is close to 1, and the *P* value of 0.000 is less than the default significance level of 0.05. We emphasize that a significant linear regression relationship exists between the predicted *F_v_*/*F_m_* from ChlF signal without dark adaptation and the *F_v_*/*F_m_* with dark adaptation. Our data clearly show that the LSSVM model is highly effective in predicting *F_v_*/*F_m_* from ChlF measured without dark adaptation.

## Discussion

Understanding the physiological mechanism of plant genetic phenotype is of great significance for improving the growth and yield of crops. ChlF is a very useful phenotypic tool for plant phenotyping and photosynthesis, and the *F_v_*/*F_m_* is subject to genetic control. The genetic phenotype of ChlF parameters is affected under stress conditions. It is very important to study the correlation between the internal difference of *F_v_*/*F_m_* among different varieties and the growth and yield of crops.

Dark adaptation has been the usual treatment before ChlF induction measurement, and it can often be used as a reference for plant stress research. Papageorgiou et al*.* [43] reported that different dark adaptation times had an important impact on the ChlF results. In addition, dark adaptation needs additional equipment and is very time-consuming. In this work, ChlF signals measured without dark adaptation have been used to obtain true *F_v_*/*F_m_* successfully by using an LSSVM model.

The experiments in this work involved the use of 6 different genetic varieties of plants, 4 levels of drought stress conditions, several different environmental temperatures (8 to 40 °C), 3 different growing seasons (spring, summer, and winter), wide range of PPFD (between 3 and 1,960 μmol photons m^−2^ s^−1^), and 3 different measured locations (wild field, greenhouse, and laboratory) (Table 1). All of the above lead to enormous differences in the ChlF parameters under a large variety of physiological conditions among different plants under different conditions (Table 2). As is well known, *F_v_*/*F_m_* is closely related to physiological status of plants. Our results clearly show that the developed model predicts the *F_v_*/*F_m_* among different samples with only very small errors (Tables 3 to 5). These data clearly prove that the LSSVM model can indeed discern the hidden relationship between ChlF signal without dark adaptation and *F_v_*/*F_m_* values with good robustness.

**Table 1.** Plant samples and experiment specifics (the light intensity of exciting the ChlF was 2,400 μmol photons m^−2^ s^−1^ for all experimental samples).

Plant and treatment	Symbol	Number of samples	Measurement location	Measurement date	Ambient temperature	Ambient PPFD
All plant samples	P	7,231	---	---	---	---
All rice samples	A	4,896	---	---	---	---
All *Osmanthus* sp. samples (*Osmanthus* sp. samples in summer and winter)	B	616	---	---	---	---
Rice without drought treatment	A0	1,335	Laboratory	July and August 2019	Between 30 and 36 °C	Between 3 and 7 μmol photons m^−2^ s^−1^
Rice with 1 h of drought treatment	A1	1,093	Laboratory	July and August 2019	Between 30 and 36 °C	Between 3 and 7 μmol photons m^−2^ s^−1^
Rice with 2 h of drought treatment	A2	1,322	Laboratory	July and August 2019	Between 30 and 36 °C	Between 3 and 7 μmol photons m^−2^ s^−1^
Rice with 4 h of drought treatment	A3	1,146	Laboratory	July and August 2019	Between 30 and 36 °C	Between 3 and 7 μmol photons m^−2^ s^−1^
*Osmanthus* sp. in summer	B1	237	Wild field	July 2021	About 33 °C	Between 58 and 1,960 μmol photons m^−2^ s^−1^
*Osmanthus* sp. in winter	B2	379	Wild field	December 2021	Between 8 and 15 °C	between 78 and 1,380 μmol photons m^−2^ s^−1^
*Euonymus japonicus Thunb*	C	314	Laboratory	April 2021	About 23 °C	About 5 μmol photons m^−2^ s^−1^
*Camellia japonica*	D	314	Laboratory	April 2021	About 23 °C	About 5 μmol photons m^−2^ s^−1^
*Capsicum annuum*	E	356	Greenhouse	August 2021	Between 36 and 40 °C	Between 58 and 1,770 μmol photons m^−2^ s^−1^
*Cerasus lannesiana* var*. speciosa*	F	335	Wild field	July 2021	About 33 °C	Between 58 and 1,960 μmol photons m^−2^ s^−1^
*Camellia japonica*	G	400	Wild field	December 2021	Between 8 and 15 °C	Between 78 and 1,380 μmol photons m^−2^ s^−1^

**Table 2.** Statistical analysis of the *F*_*v*_/*F*_*m*_ measured with dark adaptation or without dark adaptation for different samples (the results are presented as mean ± SD).

Sample type ^a^	Without dark adaptation	With dark adaptation	Sample type	Without dark adaptation	With dark adaptation
P	0.786 ± 0.051	0.818 ± 0.036	B1	0.803 ± 0.017ab	0.837 ± 0.016a
A	0.803 ± 0.012	0.831 ± 0.010	B2	0.735 ± 0.115f	0.783 ± 0.076d
B	0.761 ± 0.097	0.804 ± 0.066	C	0.746 ± 0.043e	0.780 ± 0.044b
A0	0.807 ± 0.012a	0.831 ± 0.013a	D	0.778 ± 0.018d	0.807 ± 0.018c
A1	0.805 ± 0.010ab	0.834 ± 0.007a	E	0.706 ± 0.047h	0.768 ± 0.027f
A2	0.803 ± 0.011b	0.830 ± 0.009a	F	0.801 ± 0.014bc	0.834 ± 0.009a
A3	0.798 ± 0.013c	0.828 ± 0.010b	G	0.713 ± 0.096g	0.755 ± 0.058g

^a^ P: all plant samples; A: all the rice samples; B: all *Osmanthus* sp. samples; A0: rice without drought treatment; A1: rice with 1 h of drought treatment; A2: rice with 2 h of drought treatment; A3: rice with 4 h of drought treatment; B1: *Osmanthus* sp. in summer; B2: *Osmanthus* sp. in winter; C: *Euonymus japonicus Thunb* in the laboratory; D: *Camellia japonica* in the laboratory; E: *Capsicum annuum*; F: *Cerasus lannesiana* var*. speciosa*; G: *Camellia japonica* in wild field.

**Table 3.** LSSVM model performance evaluation index $\bar{\mathrm{CC}}$ in determining *F*_*v*_/*F*_*m*_ values of training dataset from ChlF without dark adaptation under different training dataset sample numbers (10%, 20%, …, and 90% of the total sample size).

Sample type ^a^	10%	20%	30%	40%	50%	60%	70%	80%	90%
P	0.973	0.975	0.975	0.974	0.974	0.975	0.974	0.976	0.976
A	0.772	0.788	0.808	0.808	0.805	0.809	0.818	0.821	0.825
B	0.974	0.975	0.971	0.970	0.971	0.973	0.973	0.974	0.975
A0	0.677	0.720	0.745	0.736	0.739	0.739	0.792	0.792	0.765
A1	0.865	0.875	0.882	0.879	0.888	0.886	0.891	0.889	0.891
A2	0.778	0.783	0.817	0.821	0.814	0.812	0.822	0.815	0.824
A3	0.867	0.871	0.891	0.887	0.881	0.896	0.895	0.899	0.901
B1	0.851	0.920	0.939	0.961	0.944	0.962	0.968	0.970	0.970
B2	0.970	0.970	0.966	0.965	0.966	0.968	0.968	0.970	0.970
C	0.986	0.987	0.989	0.985	0.985	0.986	0.984	0.986	0.987
D	0.957	0.968	0.969	0.969	0.968	0.967	0.966	0.967	0.965
E	0.940	0.969	0.958	0.946	0.946	0.944	0.940	0.958	0.959
F	0.809	0.888	0.874	0.857	0.876	0.890	0.865	0.877	0.887
G	0.968	0.972	0.972	0.969	0.970	0.968	0.968	0.969	0.970

^a^ P: all plant samples; A: all rice samples; B: all *Osmanthus* sp. samples; A0: rice without drought treatment; A1: rice with 1 h of drought treatment; A2: rice with 2 h of drought treatment; A3: rice with 4 h of drought treatment; B1: *Osmanthus* sp. in summer; B2: *Osmanthus* sp. in winter; C: *Euonymus japonicus Thunb* in the laboratory; D: *Camellia japonica* in the laboratory; E: *Capsicum annuum*; F: *Cerasus lannesiana* var*. speciosa*; G: *Camellia japonica* in wild field.

**Table 4.** LSSVM model performance evaluation index $\bar{\text{RMSE}}$ in determining *F*_*v*_/*F*_*m*_ values of training dataset from ChlF without dark adaptation under different training dataset sample numbers (10%, 20%, …, and 90% of the total sample size).

Sample type ^a^	10%	20%	30%	40%	50%	60%	70%	80%	90%
P	0.008	0.008	0.008	0.008	0.008	0.008	0.008	0.008	0.008
A	0.006	0.006	0.006	0.006	0.006	0.006	0.006	0.006	0.006
B	0.013	0.015	0.015	0.015	0.016	0.015	0.015	0.015	0.015
A0	0.009	0.009	0.009	0.009	0.009	0.009	0.008	0.008	0.008
A1	0.004	0.004	0.004	0.004	0.004	0.004	0.004	0.004	0.004
A2	0.005	0.006	0.005	0.005	0.005	0.005	0.005	0.005	0.005
A3	0.005	0.005	0.005	0.005	0.005	0.004	0.004	0.004	0.004
B1	0.005	0.005	0.004	0.005	0.004	0.004	0.004	0.004	0.004
B2	0.017	0.019	0.019	0.019	0.020	0.019	0.019	0.018	0.018
C	0.009	0.007	0.007	0.008	0.008	0.008	0.008	0.007	0.007
D	0.005	0.005	0.005	0.005	0.005	0.005	0.005	0.005	0.005
E	0.008	0.007	0.007	0.009	0.009	0.008	0.009	0.007	0.007
F	0.006	0.005	0.005	0.005	0.005	0.005	0.005	0.005	0.005
G	0.015	0.014	0.015	0.016	0.015	0.016	0.016	0.015	0.015

^a^ P: all plant samples; A: all rice samples; B: all *Osmanthus* sp. samples; A0: rice without drought treatment; A1: rice with 1 h of drought treatment; A2: rice with 2 h of drought treatment; A3: rice with 4 h of drought treatment; B1: *Osmanthus* sp. in summer; B2: *Osmanthus* sp. in winter; C: *Euonymus japonicus Thunb* in the laboratory; D: *Camellia japonica* in the laboratory; E: *Capsicum annuum*; F: *Cerasus lannesiana* var*. speciosa*; G: *Camellia japonica* in wild field.

**Table 5.** LSSVM model performance evaluation index $\bar{\mathrm{RPD}}$ in determining *F*_*v*_/*F*_*m*_ values of training dataset from ChlF without dark adaptation under different training dataset sample numbers (10%, 20%, …, and 90% of the total sample size).

Sample type ^a^	10%	20%	30%	40%	50%	60%	70%	80%	90%
P	4.577	4.500	4.519	4.404	4.457	4.490	4.493	4.600	4.652
A	1.637	1.632	1.700	1.706	1.689	1.704	1.743	1.749	1.770
B	4.727	4.530	4.238	4.170	4.257	4.334	4.355	4.454	4.501
A0	1.416	1.420	1.472	1.460	1.453	1.459	1.602	1.611	1.524
A1	1.767	1.778	1.817	1.850	1.854	1.862	1.900	1.897	1.920
A2	1.725	1.679	1.768	1.769	1.714	1.694	1.729	1.687	1.720
A3	2.042	2.053	2.230	2.153	2.111	2.236	2.225	2.261	2.297
B1	2.454	3.371	3.278	3.723	3.317	3.625	3.934	4.021	4.163
B2	4.422	4.170	3.919	3.851	3.910	3.990	4.007	4.101	4.131
C	6.253	6.001	6.462	5.489	5.584	5.846	5.590	6.039	6.235
D	3.344	3.749	3.725	3.728	3.629	3.622	3.504	3.549	3.479
E	3.875	4.422	3.676	3.219	3.304	3.500	3.370	3.683	3.929
F	1.525	1.909	1.816	1.637	1.815	1.963	1.753	1.865	1.973
G	4.017	4.052	4.056	3.734	3.847	3.785	3.755	3.854	3.892

^a^ P: all plant samples; A: all rice samples; B: all *Osmanthus* sp. samples; A0: rice without drought treatment; A1: rice with 1 h of drought treatment; A2: rice with 2 h of drought treatment; A3: rice with 4 h of drought treatment; B1: *Osmanthus* sp. in summer; B2: *Osmanthus* sp. in winter; C: *Euonymus japonicus Thunb* in the laboratory; D: *Camellia japonica* in the laboratory; E: *Capsicum annuum*; F: *Cerasus lannesiana* var*. speciosa*; G: *Camellia japonica* in wild field.

**Table 6.** LSSVM model performance evaluation index $\bar{\mathrm{CC}}$ in determining *F*_*v*_/*F*_*m*_ values of test dataset from ChlF without dark adaptation under different training dataset sample numbers (10%, 20%, …, and 90% of the total sample size).

Sample type ^a^	10%	20%	30%	40%	50%	60%	70%	80%	90%
P	0.947	0.955	0.958	0.960	0.961	0.962	0.963	0.964	0.962
A	0.706	0.751	0.765	0.757	0.779	0.780	0.772	0.806	0.802
B	0.959	0.965	0.966	0.967	0.967	0.966	0.966	0.972	0.969
A0	0.570	0.645	0.662	0.652	0.683	0.688	0.690	0.763	0.762
A1	0.841	0.857	0.865	0.872	0.872	0.872	0.871	0.888	0.892
A2	0.743	0.758	0.773	0.757	0.778	0.799	0.765	0.841	0.831
A3	0.817	0.859	0.865	0.864	0.884	0.866	0.869	0.880	0.882
B1	0.907	0.934	0.953	0.939	0.958	0.950	0.949	0.917	0.837
B2	0.953	0.959	0.960	0.962	0.962	0.960	0.960	0.966	0.964
C	0.946	0.939	0.963	0.961	0.962	0.954	0.973	0.974	0.939
D	0.956	0.959	0.962	0.963	0.965	0.961	0.963	0.958	0.962
E	0.674	0.655	0.647	0.699	0.746	0.783	0.771	0.810	0.838
F	0.777	0.776	0.790	0.829	0.800	0.786	0.788	0.876	0.847
G	0.955	0.958	0.959	0.962	0.958	0.962	0.961	0.961	0.963

^a^ P: all plant samples; A: all rice samples; B: all *Osmanthus* sp. samples; A0: rice without drought treatment; A1: rice with 1 h of drought treatment; A2: rice with 2 h of drought treatment; A3: rice with 4 h of drought treatment; B1: *Osmanthus* sp. in summer; B2: *Osmanthus* sp. in winter; C: *Euonymus japonicus Thunb* in the laboratory; D: *Camellia japonica* in the laboratory; E: *Capsicum annuum*; F: *Cerasus lannesiana* var*. speciosa*; G: *Camellia japonica* in wild field.

**Table 7.** LSSVM model performance evaluation index $\bar{\text{RMSE}}$ in determining *F*_*v*_/*F*_*m*_ values of test dataset from ChlF without dark adaptation under different training dataset sample numbers (10%, 20%, …, and 90% of the total sample size).

Sample type ^a^	10%	20%	30%	40%	50%	60%	70%	80%	90%
P	0.012	0.011	0.011	0.010	0.010	0.010	0.010	0.010	0.010
A	0.007	0.007	0.007	0.007	0.006	0.006	0.007	0.006	0.006
B	0.019	0.018	0.017	0.017	0.017	0.017	0.017	0.016	0.017
A0	0.011	0.010	0.010	0.010	0.009	0.009	0.009	0.009	0.009
A1	0.004	0.004	0.004	0.004	0.004	0.004	0.004	0.004	0.004
A2	0.006	0.006	0.006	0.006	0.006	0.006	0.006	0.005	0.005
A3	0.006	0.005	0.005	0.005	0.005	0.005	0.005	0.005	0.005
B1	0.007	0.006	0.005	0.005	0.005	0.005	0.005	0.005	0.005
B2	0.024	0.022	0.022	0.022	0.021	0.021	0.021	0.020	0.021
C	0.015	0.015	0.012	0.013	0.012	0.012	0.010	0.010	0.017
D	0.006	0.005	0.005	0.005	0.005	0.005	0.005	0.005	0.006
E	0.025	0.023	0.023	0.002	0.019	0.017	0.018	0.021	0.018
F	0.012	0.008	0.007	0.006	0.007	0.007	0.006	0.007	0.006
G	0.019	0.018	0.018	0.017	0.017	0.017	0.016	0.017	0.017

^a^ P: all plant samples; A: all rice samples; B: all *Osmanthus* sp. samples; A0: rice without drought treatment; A1: rice with 1 h of drought treatment; A2: rice with 2 h of drought treatment; A3: rice with 4 h of drought treatment; B1: *Osmanthus* sp. in summer; B2: *Osmanthus* sp. in winter; C: *Euonymus japonicus Thunb* in the laboratory; D: *Camellia japonica* in the laboratory; E: *Capsicum annuum*; F: *Cerasus lannesiana* var*. speciosa*; G: *Camellia japonica* in wild field.

**Table 8.** LSSVM model performance evaluation index $\bar{\mathrm{RPD}}$ in determining *F*_*v*_/*F*_*m*_ values of test dataset from ChlF without dark adaptation under different training dataset sample numbers (10%, 20%, …, and 90% of the total sample size).

Sample type ^a^	10%	20%	30%	40%	50%	60%	70%	80%	90%
P	3.095	3.362	3.468	3.566	3.626	3.689	3.706	3.757	3.724
A	1.402	1.511	1.552	1.528	1.600	1.604	1.581	1.692	1.695
B	3.491	3.74	3.87	3.901	3.941	3.918	3.884	4.209	4.176
A0	1.187	1.300	1.322	1.309	1.362	1.365	1.376	1.556	1.548
A1	1.627	1.672	1.763	1.753	1.803	1.780	1.794	1.884	1.925
A2	1.469	1.504	1.547	1.506	1.581	1.652	1.549	1.837	1.831
A3	1.702	1.910	1.985	1.971	2.130	1.987	2.050	2.110	2.272
B1	2.330	2.790	3.292	2.960	3.371	3.282	3.158	3.071	2.507
B2	3.245	3.466	3.556	3.589	3.635	3.608	3.569	3.849	3.858
C	3.127	3.006	3.700	3.625	3.670	3.802	4.744	4.933	4.141
D	3.179	3.256	3.299	3.441	3.364	3.285	3.528	3.079	3.336
E	1.168	1.189	1.184	1.311	1.387	1.578	1.451	2.104	1.726
F	0.926	1.165	1.336	1.454	1.293	1.271	1.334	1.254	1.512
G	3.086	3.240	3.241	3.377	3.222	3.414	3.497	3.462	3.520

^a^ P: all plant samples; A: all rice samples; B: all *Osmanthus* sp. samples; A0: rice without drought treatment; A1: rice with 1 h of drought treatment; A2: rice with 2 h of drought treatment; A3: rice with 4 h of drought treatment; B1: *Osmanthus* sp. in summer; B2: *Osmanthus* sp. in winter; C: *Euonymus japonicus Thunb* in the laboratory; D: *Camellia japonica* in the laboratory; E: *Capsicum annuum*; F: *Cerasus lannesiana* var*. speciosa*; G: *Camellia japonica* in wild field.

The computation time for each test sample is less than 4 ms (processor: Intel Core i5-9400F CPU @ 2.90GHz) and much less than the dark-adaptation time (almost 20 min) taken in the traditional experiments. The machine learning method proved effective in uncovering the hidden relationships between ChlF signals of plant leaves with and without dark adaptation. The ability to measure *F_v_*/*F_m_* without dark adaptation will save experimental time and cost. More important, this will allow *F_v_*/*F_m_* to be used in the field and in real time, which will make *F_v_*/*F_m_* a much more convenient measure in probing the physiological status of plants. This work provides a high-throughput method for determining the important photosynthetic feature through ChlF, which would provide plant physiological features in phenotyping.

This work also implies that the hidden nonlinear biological photosynthetic behavior can be discerned by artificial intelligence. The concept in this work is not only limited to predicting *F_v_*/*F_m_*, but it may be also used to predict other ChlF parameters, such as effective photochemical quantum yield of PSII (Y[II]), quantum yield of regulated energy dissipation in PSII (Y[NPQ]), and quantum yield of nonregulated heat energy dissipation and fluorescence emission (Y[NO]) after model retraining.

Recently, there have been many updated deep learning networks in the literature [44], such as Extreme Gradient Boosting (XGboost) [45] and Light Gradient Boosting Machine (LightGBM) [46]. The performance of XGboost and LightGBM were tested for predicting *F_v_*/*F_m_* values from ChlF measurements without dark adaptation in this work for comparison, but their performance is similar to the LSSVM model, which implies that an LSSVM model is enough for this application. In this work, we thus report only the results from the simple LSSVM model as its performance is already very promising. The LSSVM model, used here, has shown great promise with small prediction errors, but, as is the case for other neural network-based tools, more experiments are needed to build a much bigger public training and testing dataset like the well-known imageNet for human face recognition [47] to call for improvements of the prediction model.

Dark adaptation of photosynthetic samples has been essential in measuring quantum yield of PSII via *F_v_*/*F_m_* through ChlF-based analysis of photosynthesis and plant responses. We developed an LSSVM model that can obtain *F_v_*/*F_m_* from ChlF signals measured without dark adaptation. The model was validated with data collected from many different plants under varied conditions. Our results have established that the LSSVM model could indeed determine *F_v_*/*F_m_* from ChlF measurements without dark adaptation. We emphasize that this work demonstrates that *F_v_*/*F_m_* can be determined without dark adaptation of plants, which will make the measurement more convenient and enhance the research of plant physiology and phenotyping.

## Data Availability

Data are available on request from the authors.
